# Supplementary material for: In Silico Screening for Pesticide Candidates against the Desert Locust Schistocerca gregaria
Source: Life (Basel). 2022 Mar 7;12(3):387. doi: 10.3390/life12030387 (PMC8953258; doi:10.3390/life12030387)
Supplement: Supplementary file 1 [file life-12-00387-s001.zip › life-1608568-supplementary.pdf]

life

MDPI

Article

In Silico Screening for Pesticide Candidates against the Desert Locust *Schistocerca gregaria*

Graham E. Jackson [1,*], Gerd Gäde [2] and Heather G. Marco [2]

[1] Department of Chemistry, University of Cape Town, Cape Town 7701, South Africa
[2] Department of Biological Sciences, University of Cape Town, Cape Town 7701, South Africa; gerd.gade@uct.ac.za (G.G); heather.marco@uct.ac.za (H.G.M.)
* Correspondence: graham.jackson@uct.ac.za; Tel.: +27-216-502-531

Abstract: Adipokinetic hormone (AKH) is one of the most important metabolic neuropeptides in insects, with actions similar to glucagon in vertebrates. AKH regulates carbohydrate and fat metabolism by mobilizing trehalose and diacylglycerol into circulation from glycogen and triacylglycerol stores, respectively, in the fat body. The short peptide (8 to 10 amino acids long) exerts its function by binding to a rhodopsin-like G protein-coupled receptor located in the cell membrane of the fat body. The AKH receptor (AKHR) is, thus, a potential target for the development of novel specific (peptide) mimetics to control pest insects, such as locusts, which are feared for their prolific breeding, swarm-forming behavior and voracious appetite. Previously, we proposed a model of the interaction between the three endogenous AKHs of the desert locust, *Schistocerca gregaria*, and the cognate AKHR (Jackson et al., Peer J. 7, e7514, 2019). In the current study we have performed in silico screening of two databases (NCI Open 2012 library and Zinc20) to identify compounds which may fit the endogenous Schgr-AKH-II binding site on the AKHR of *S. gregaria*. In all, 354 compounds were found to fit the binding site with glide scores < -8. Using the glide scores and binding energies, 7 docked compounds were selected for molecular dynamic simulation in a phosphatidylcholine membrane. Of these 7 compounds, 4 had binding energies which would allow them to compete with Schgr-AKH-II for the receptor binding site and so are proposed as agonistic ligand candidates. One of the ligands, ZINC000257251537, was tested in a homospecific in vivo biological assay and found to have significant antagonistic activity.

Keywords: adipokinetic hormone; desert locust; *Schistocerca gregaria*; in silico screening; ZINC20

Citation: Jackson, G.E.; Gäde, G.; Marco, H.G. In Silico Screening for Pesticide Candidates against the Desert Locust *Schistocerca gregaria*. *Life* **2022**, *12*, 387. https://doi.org/10.3390/life12030387

Academic Editors: Rossella Miele and Roberta Lattanzi

Received: 7 February 2022
Accepted: 5 March 2022
Published: 7 March 2022

Publisher's Note: MDPI stays neutral with regard to jurisdictional claims in published maps and institutional affiliations.

Copyright: © 2022 by the authors. Licensee MDPI, Basel, Switzerland. This article is an open access article distributed under the terms and conditions of the Creative Commons Attribution (CC BY) license (http://creativecommons.org/licenses/by/4.0/).

Supplementary Materials:

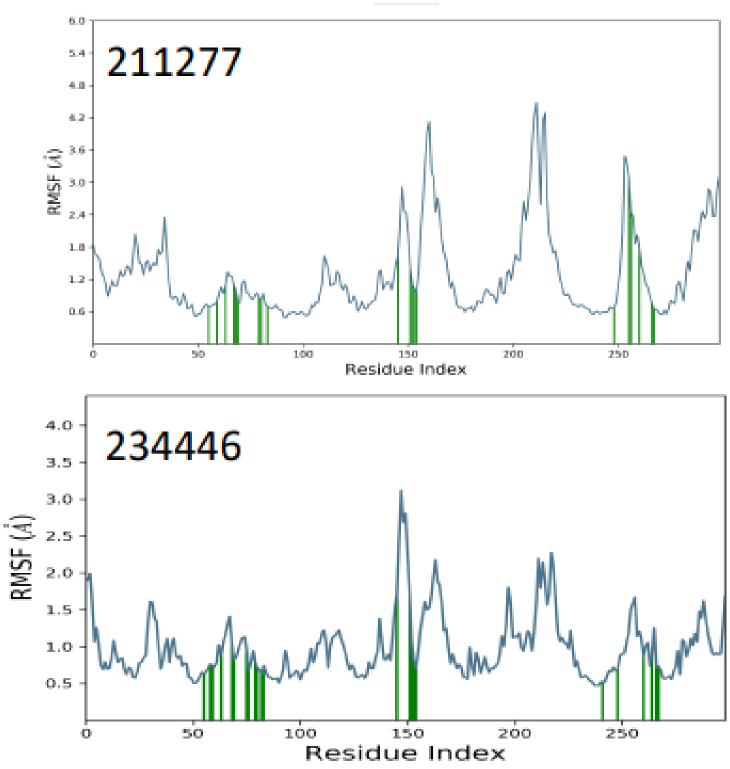

Figure S1. Protein Root Mean Square Fluctuation (RMSF) of Cα a during 50 ns MD simulation in POPC membrane.

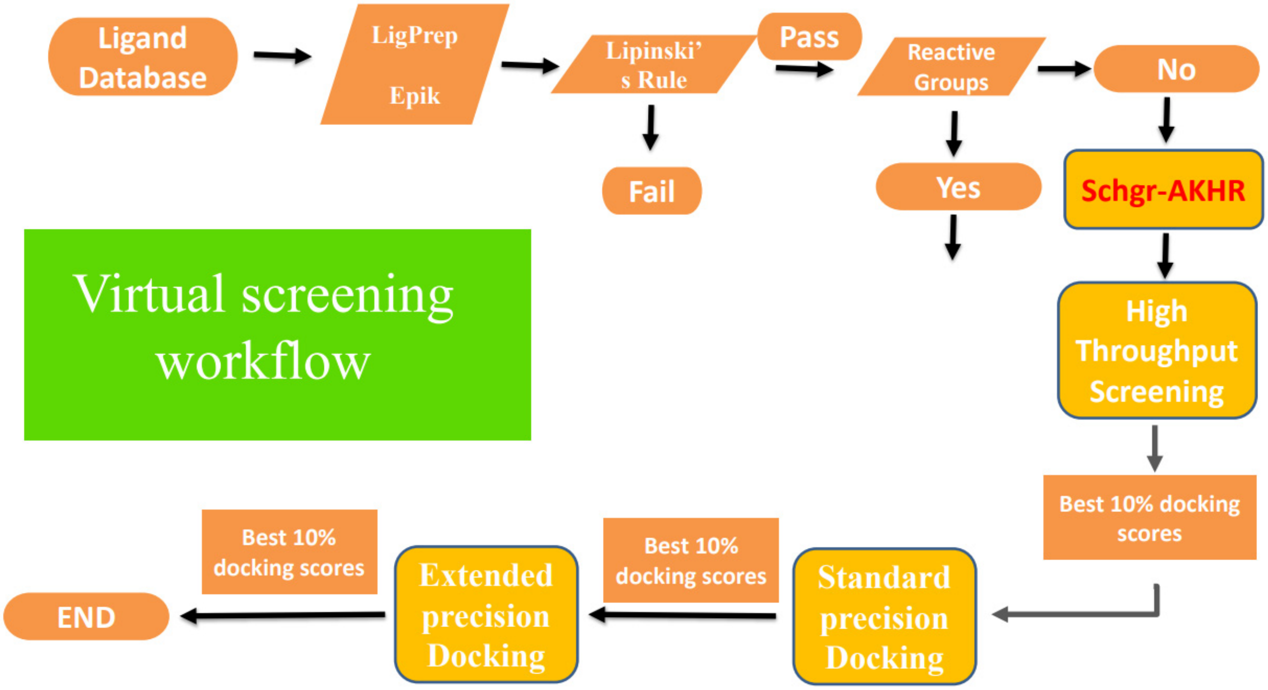

Figure S2. Virtual screening workflow.
